# Alterations of perineuronal nets in the dorsolateral prefrontal cortex of neuropsychiatric patients

**DOI:** 10.1186/s40345-019-0161-0

**Published:** 2019-11-15

**Authors:** Julia Alcaide, Ramón Guirado, Carlos Crespo, José Miguel Blasco-Ibáñez, Emilio Varea, Julio Sanjuan, Juan Nacher

**Affiliations:** 1 Neurobiology Unit, Department of Cell Biology, Interdisciplinary Research Structure for Biotechnology and Biomedicine (BIOTECMED), Universitat de Valencia, Dr. Moliner 50, 46100 Burjassot, Spain; 2 Department of Medicine, Universitat de València, Valencia, Spain; 3 CIBERSAM: Spanish National Network for Research in Mental Health, Madrid, Spain; 4 Fundación Investigación Hospital Clínico de Valencia, INCLIVA, Valencia, Spain

**Keywords:** Schizophrenia, Bipolar disorder, Major depression, Perineuronal nets, Parvalbumin, Prefrontal cortex

## Abstract

**Background:**

Alterations in the structure and physiology of interneurons in the prefrontal cortex (PFC) are important factors in the etiopathology of different psychiatric disorders. Among the interneuronal subpopulations, parvalbumin (PV) expressing cells appear to be specially affected. Interestingly, during development and adulthood the connectivity of these interneurons is regulated by the presence of perineuronal nets (PNNs), specialized regions of the extracellular matrix, which are frequently surrounding PV expressing neurons. Previous reports have found anomalies in the density of PNNs in the PFC of schizophrenic patients. However, although some studies have described alterations in PNNs in some extracortical regions of bipolar disorder patients, there are no studies focusing on the prefrontocortical PNNs of bipolar or major depression patients. For this reason, we have analyzed the density of PNNs in post-mortem sections of the dorsolateral PFC (DLPFC) from the Stanley Neuropathology Consortium, which includes controls, schizophrenia, bipolar and major depression patients.

**Results:**

We have not observed differences in the distribution of PV+ cells or PNNs, or in the percentage of PV+ interneurons surrounded by PNNs. The density of PV+ interneurons was similar in all the experimental groups, but there was a significantly lower density of PNNs in the DLPFC of bipolar disorder patients and a tendency towards a decrease in schizophrenic patients. No differences were found when evaluating the density of PV+ cells surrounded by PNNs. Interestingly, when assessing the influence of demographic data, we found an inverse correlation between the density of PNNs and the presence of psychosis.

**Conclusions:**

The present results point to prefrontocortical PNNs and their role in the regulation of neuronal plasticity as putative players in the etiopathology of bipolar disorder and schizophrenia. Our findings also suggest a link between these specialized regions of the extracellular matrix and the presence of psychosis.

## Background

Psychiatric disorders affect 1 out of 4 people at some point during their lives and represent a huge economic and social burden (World Health Organization 2001). However, the neuronal mechanisms underlying the development of these diseases still remain unclear, highlighting the need for further research in basic neuroscience. Bipolar disorder, major depression or schizophrenia are very different pathologies, but they share certain structural and functional abnormalities, including alterations in the inhibitory networks of the prefrontal cortex (PFC).

These alterations are especially pronounced in the PFC of schizophrenia and bipolar disorder patients, where reductions of GAD67, one of the enzymes responsible for GABA synthesis, have been described (Akbarian et al. 1995; Guidotti et al. 2000). There is also evidence of the involvement of the prefrontocortical GABAergic system in major depression: patients show reduced GABA levels (Hasler et al. 2007) and decreased expression of GABAergic genes (Zhao et al. 2018). Moreover, schizophrenic patients display altered expression of GABA-related genes in their PFC, such as transporters or receptors of this neurotransmitter (Benes et al. 1996; Hashimoto et al. 2008; Hoftman et al. 2015; Volk et al. 2001).

Parvalbumin (PV) expressing interneurons constitute one of the three families of cortical interneurons following a classification by their calcium-binding protein expression. PV expressing interneurons effectively synchronize large populations of neurons, regulating the synaptic excitatory tone in different cortical regions (Hu et al. 2014). Furthermore, alterations in this subpopulation of interneurons have been described in different psychiatric diseases, such as major depression, schizophrenia, autism or bipolar disorder (Marín 2012). In the PFC of bipolar disorder patients there is a reduction in the expression of PV mRNA (Sibille et al. 2011). Similarly, in schizophrenic patients there is a reduction in the expression of GAD67 mRNA, specifically in PV expressing cells (Hashimoto et al. 2003) and these alterations have been related to abnormalities in the synaptic input that PV expressing interneurons establish onto pyramidal cells (Lewis et al. 2012). Although some studies on major depression patients have not found changes in PV expression or in PV+ cell density in the PFC (Beasley et al. 2002; Rajkowska et al. 2007; Sibille et al. 2011), at least one found a decrease in PV gene expression (Tripp et al. 2012). Similarly, anomalies in PV expressing interneurons have been also reported in animal models of major depression (Perova et al. 2015; Pesarico et al. 2019; Sauer et al. 2015; Todorovic et al. 2019).

Alterations of prefrontocortical PV expressing interneurons are likely to be mediated by the expression of molecules related to interneuronal plasticity. Known regulators of the structure and connectivity of PV expressing interneurons include the polysialylated form of the neural cell adhesion molecule (PSA-NCAM) (Nacher et al. 2013). In fact, we have described a reduced expression of PSA-NCAM in layers IV and V of the PFC of schizophrenic patients (Gilabert-Juan et al. 2012).

Other important regulators of interneuronal plasticity and particularly of PV expressing interneurons are the perineuronal nets (PNNs), specialized structures of the extracellular matrix, which predominantly surround the soma and proximal neurites of PV expressing interneurons (Sorg et al. 2016). PNNs are composed by hyaluronic acid, tenascin-R, link proteins and proteoglycans (mainly chondroitin sulphate proteoglycans) (Kwok et al. 2011). Although the functions of PNNs are not clear yet, it is known that they mediate the closure of the critical periods of cortical plasticity through the stabilization of recently formed synapses and the prevention of synaptogenesis (McRae and Porter 2012; Pizzorusso et al. 2002; Wang and Fawcett 2012). PNNs are also important to maintain the local homeostasis of ions (Morawski et al. 2015) and to protect neurons against oxidative stress (Cabungcal et al. 2013). Particularly, PNNs control the activity and excitability of PV expressing neurons (Balmer 2016; Dityatev et al. 2007; Favuzzi et al. 2017). In fact, the enzymatic attenuation of PNNs alters the excitatory input to fast-spiking PV expressing interneurons (Hayani et al. 2018). Moreover, alterations in the glycoprotein tenascin-R, a key component of the PNNs, which helps in the stabilization of these regions of the extracellular matrix, impairs the perisomatic inhibitory input to pyramidal neurons (Saghatelyan et al. 2000, 2001, 2003).

Interestingly, abnormalities of chondroitin sulfate proteoglycans have been implicated in the etiopathology of different psychiatric disorders (Berretta 2012). In this line, some studies have shown that subjects with schizophrenia had a reduced density of PNNs in the PFC (Enwright et al. 2016; Mauney et al. 2013) and similar reductions have been found in different animal models of this disorder (Castillo-Gómez et al. 2017; Matuszko et al. 2017; Paylor et al. 2016). Although there are some reports describing alterations in PNNs in certain extracortical regions of bipolar disorder patients (Pantazopoulos et al. 2010; Steullet et al. 2018), to our knowledge there are no studies to date focusing on the PFC of bipolar disorder or major depression patients.

In this study, we investigated whether the densities of PV expressing interneurons and PNNs were affected in the DLPFC of individuals suffering from major depression, schizophrenia or bipolar disorder. We have selected this prefrontocortical region because structural and functional alterations have already been found in these diseases (Phillips et al. 2003). Moreover, previous studies in the DLPFC, including our own (Gilabert-Juan et al. 2012), have found changes in molecules related to inhibitory neurotransmission and in PV+ cells and PNNs, specifically in Brodmann area 9 (BA9). We have performed histochemical analysis in brain sections from post-mortem samples of psychiatric patients and a control group and have measured the density of PV and PNN positive cells and their co-localization.

## Methods

### Samples and histological processing

Frozen 14 µm thick coronal sections containing the dorsolateral prefrontal cortex (DLPFC) of patients diagnosed with major depression, bipolar disorder or schizophrenia, and control subjects were obtained from the Stanley Medical Research Institute (Bethesda, MD, USA). All patient records were reviewed by one psychiatrist and summarized in narrative form, and the information was entered into a computerized database by identifying number only. When all the information was collected, a DSM-IV psychiatric diagnosis was made independently by two senior psychiatrists. If there was disagreement between them, the records were given to a third senior psychiatrist and a consensus diagnosis was established (Torrey et al. 2000). The cohort consists of 15 individuals in each group, although some samples were not analyzed due to the poor condition of the tissue. The demographic and recruitment data of patients have been described earlier (Gilabert-Juan et al. 2012) and are summarized in Table 1. All brains underwent clinical neuropathological examination by two neuropathologists, none demonstrated evidence of neurodegenerative changes or other pathological lesions.

**Table 1:** Clinical and demographical data of the Stanley Foundation Neuropathology Consortium

	Control	MD	BD	SCHZ
n	11	11	13	10
Age, years, mean (SEM), (range)	46.6 (29–59)	48 (32–65)	42.9 (25–57)	43.5 (25–62)
Sex male/female	4/7	5/6	5/8	5/5
PMI, hours, mean (SEM), (range)	26 (8–42)	25.8 (7–47)	31.5 (13–62)	35.4 (12–60)
Tissue pH, mean (SEM), (range)	6.3 (5.8–6.6)	6.2 (5.8–6.5)	6.2 (5.8–6.5)	6.2 (5.8–6.6)
Suicide	0	4	8	3
Alcohol use	7	7	9	6
Psychosis	0	0	10	10
Brain weight, g, mean (SEM), (range)	1529.5 (1305–1840)	1439.1 (1240–1740)	1444.5 (1130–1690)	1465 (1270–1640)
Duration, years, mean (SEM), (range)		13.4 (2–42)	19 (6–43)	22.1 (5–45)

Sections were thawed and immediately fixed by immersion in a solution of paraformaldehyde 4% during 20 min. After fixation, sections were washed in phosphate buffer (PB: 0.1 M, pH 7.4) and processed immediately for immunohistochemistry. All the sections studied passed through the procedures simultaneously, to minimize any difference from the immunohistochemical protocols themselves. Experimenters were blind to the experimental condition until the end of the quantification.

### Histochemistry

Tissue was processed for fluorescence histochemistry as follows. After PB washing, slices were incubated in 10% normal donkey serum (NDS), 0.2% Triton-X100 (Sigma) in phosphate buffered saline (PBS) for 1 h. Then, slices were washed again in PBS and incubated for 48 h at 4 °C with polyclonal guinea pig anti-PV antibody (1:2000, Synaptic Systems, Gottingen, Germany) and biotin-conjugated Wisteria floribunda agglutinin (WFA, 1:200, Sigma) diluted in PBS 0.2% Triton-X100. After washing with PBS, sections were incubated at room temperature for 2 h with the secondary antibody: A555-conjugated goat anti-guinea pig (1:400, LifeTechnologies, Carlsbad, CA, USA) and A647-conjugated streptavidin (1:400, LifeTechnologies, Carlsbad, CA, USA), both diluted in PBS 0.2% Triton-X100. Finally, sections were washed in PB, mounted on slides and coverslipped using fluorescence mounting medium (Dako, Glostrup, Denmark).

### Confocal microscopy and image analyses

In order to analyze the densities of both PV positive interneurons and PNNs, single confocal planes were obtained using a laser scanning confocal microscope (Leica TCS SPE) and images were processed with FIJI (ImageJ, NIH). We analyzed three images of 275.17 μm^2^ from each patient, taken randomly in the deep layers of the DLPFC (layers III, IV, V and VI). In order to obtain these images, we delimited the total area containing the deep layers of the DLPFC and selected three squares of 275.17 μm^2^, which generated with random coordinates within this area. During this procedure we excluded the squares with areas that were partially outside the delimited region. This procedure was used for every patient and experimental group. PV expressing interneurons and PNNs were counted in all three images and their density was calculated as neurons/area.

### Statistical analyses

Group differences were assessed using a one-way ANOVA and multiple comparisons p-value adjustment by Bonferroni post hoc analysis. We first performed a Bartlett’s test to assess the homoscedasticity of the data and Shapiro test for normality. No p-value was found to be statistically significant, thus, we were able to perform parametric tests such as ANOVA. Analyses were performed with R version 3.6.1 (R Core Team 2019). and graphs created using GraphPad Prism 6. All values are expressed as mean ± standard error of the mean (SEM). The cutoff for statistical significance was set as p = 0.05. Effect of post-mortem interval (PMI), brain pH, brain weight, age, gender, suicide, substance/alcohol abuse, presence of psychosis, side of the brain (right or left hemisphere), age of disease onset or lifetime neuroleptic use (in fluphenazine mg equivalents) was assessed by univariable and multivariable analyses. First, we performed a model for each variable in the data base: categorical variables were analyzed by one-way ANOVA (age, sex, suicide, alcohol abuse, presence of psychosis, side of the brain), continuous variables (age, brain pH, brain weight, PMI, age of disease onset or lifetime neuroleptic use) were analyzed by simple linear regression analysis. Second, we performed a multivariable analysis, searching for models that could integrate all the variables. For developing the models, we used the *glmulti* library (Calcagno 2018). The method for the selection of the best model has been based on the Akaike Information Criterion (Sakamoto et al. 1986). Since the model includes categorical and continuous variables and a continuous variable as dependent variable we used an analysis of covariance (ANCOVA).

## Results

### Distribution of PNNs and PV expressing cells in the DLPFC

Although PNNs could be observed in all the extension of the DLPFC, we found a differential distribution among layers. In layer I we found virtually no PNNs. A low PNN density was found in layers II and IV. Layers V and VI had an intermediate density of PNNs and the highest PNN density was found in layer III.

**Fig. 1:**
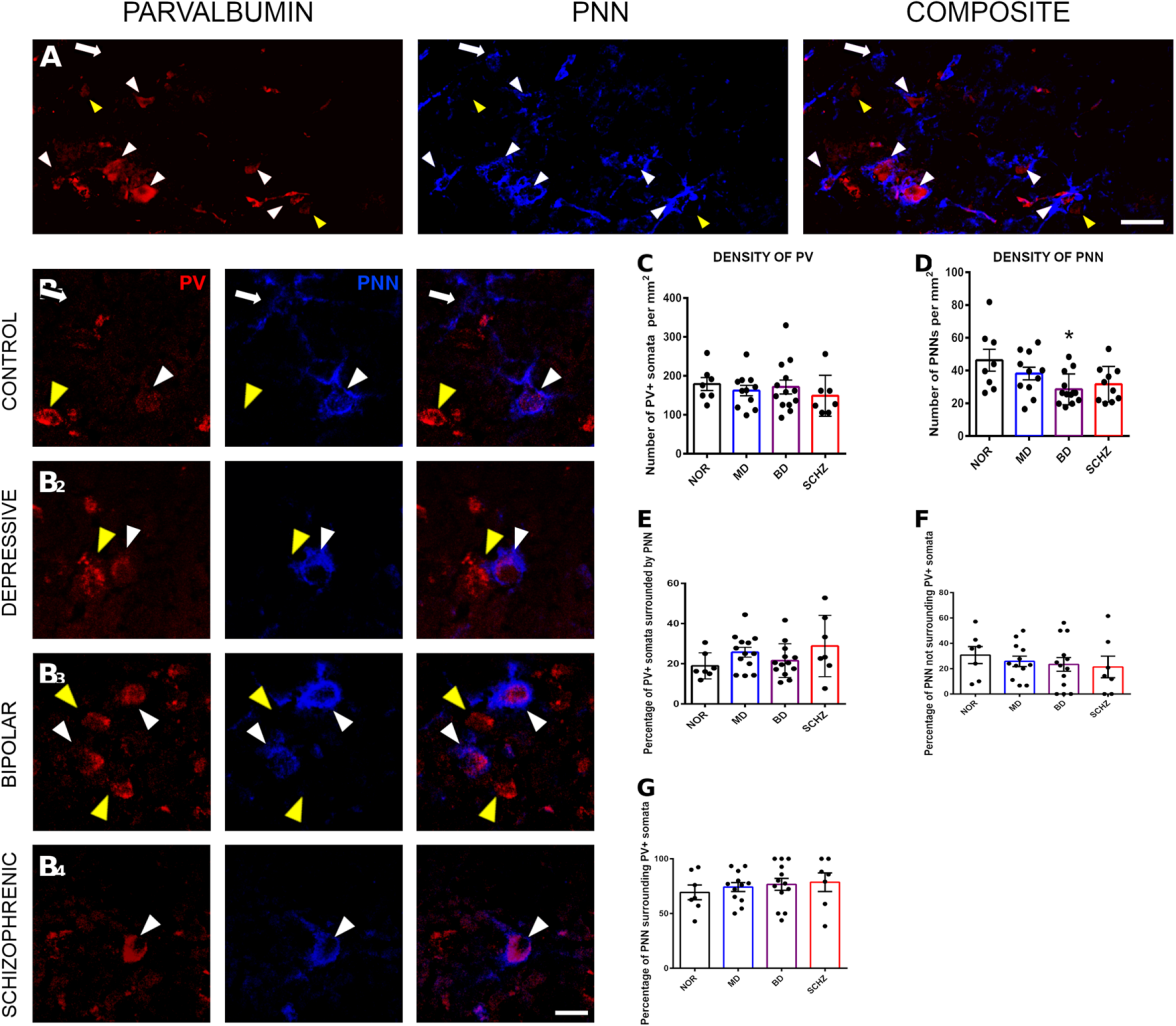
Alterations of PV and PNN densities in neuropsychiatric disorders. **a** Panoramic confocal microphotograph showing WFA-labeled PNNs (blue) surrounding PV positive somata (red) in the deep layers of the dorsolateral prefrontal cortex (DLPFC) of a control individual. **b** Images of single confocal planes showing PNNs surrounding PV+ somata in the DLPFC in patients suffering from major depression (MD), bipolar disorder (BD) and schizophrenia (SCHZ) and controls (NOR). White arrowheads point to PV+ somata surrounded by PNNs, yellow arrowheads point to PV+ cells lacking PNNs and white arrows point to PNNs surrounding PV− somata. **c**–**g** Histograms showing the density of PV expressing interneurons (**c**), PNNs (**d**) and the percentages of PV expressing somata surrounded by PNNs (**e**), of PNNs surrounding PV+ somata (**f**) and of PNNs not surrounding PV+ somata (**g**). There are significant differences in the density of PNN between control individuals and bipolar patients (*p = 0.012). Scale bar: 20 µm

The distribution of PV positive cells was, as expected, similar to that of PNNs (Fig. 1a). The highest density of PV positive cells was found in layers III and IV, followed by those in layers II and V. We found the lower density of cells in layers I and VI.

We performed all our analyses in deep layers of the DLPFC, including layers III, IV, V and VI. The percentages of PNNs surrounding PV+ somata were around 75% in every group and no significant differences were found between them (controls: 69.3 ± 6.7%; schizophrenia: 78.6 ± 8.5%; bipolar: 76.64 ± 5.4%; major depression: 74.2 ± 5,4%; F(3, 35) = 0.3965, p = 0.7563). Interestingly, some of the PV negative somata surrounded by PNN had the typical morphology of pyramidal neurons.

### Bipolar individuals show a lower PNN density in the DLPFC

We compared the density of both PNNs and PV immunoreactive neurons in the DLPFC of patient and control groups (Fig. 1b). One way-ANOVA analysis showed that there were no differences in the density of PV expressing interneurons between the four groups (F(3, 34) = 0.4334, p = 0.8099; Fig. 1c).

When analyzing the density of PNNs, we found significant differences in PNN density between the four groups (F(3, 38) = 3.581, p = 0.0225). Bonferroni’s post hoc analysis showed a significantly lower density of PNNs in patients suffering from bipolar disorder compared to the control group (F(3, 38) = 3.581, p = 0.012). We also found a tendency towards a lower density of PNNs in schizophrenic patients compared to the control group (F(3, 38) = 3.581, p = 0.0643; Fig. 1d).

Finally, we analyzed in the four groups the percentages of PV expressing interneurons surrounded by PNNs (F(3, 36) = 1.586, p = 0.1193; Fig. 1e), of PNNs surrounding PV expressing interneurons (F(3, 35) = 0.3965, p = 0.7563; Fig. 1f) and of PNNs not associated with PV+ somata (F(3, 35) = 0.3965, p = 0.7563; Fig. 1g). We did not find any significant difference between groups in any of these parameters.

### Patients with psychosis show a lower density of PNNs

**Fig. 2:**
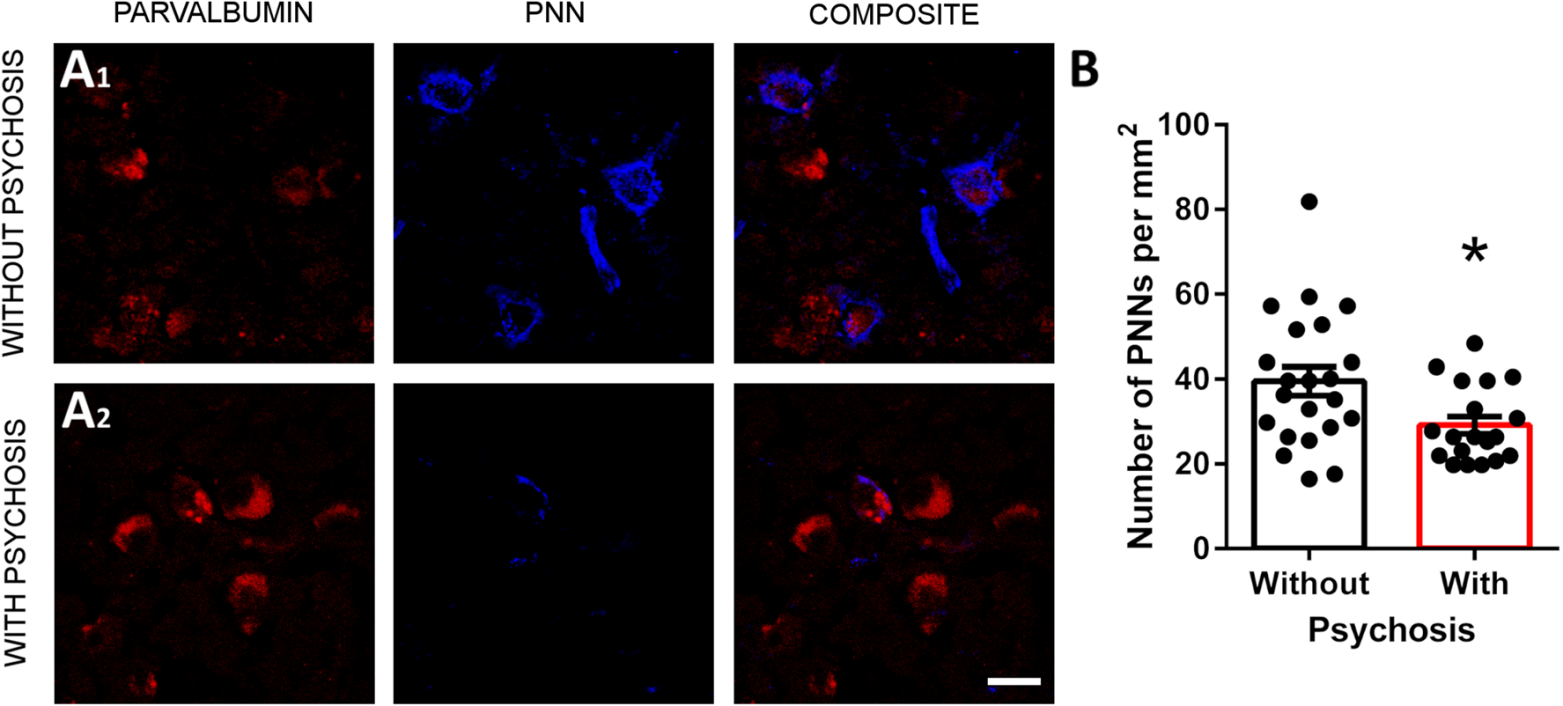
Effects of psychosis on the density of PNNs. **a** Confocal images, showing WFA-labeled PNNs (blue) surrounding PV positive somata (red) in the dorsolateral prefrontal cortex (DLPFC) of patients diagnosed with (**a2**) and without (**a1**) psychosis. **b** Histogram comparing the density of PNNs in individuals diagnosed with (black bar) or without (white bar) psychosis (Pr(> F) = 0.034; Pr(> |t|) = 0.035). Scale bar: 20 µm

We performed univariable and multivariable analyses to assess the influence of demographic data (age, sex, PMI, weight of the brain…) on our experimental conditions (see “Methods” section). We only observed an effect of the presence of psychosis: patients who had suffered psychotic episodes had lower PNN density (Fig. 2). This significant difference was found both using univariable (Pr(> F) = 0.034) and multivariable analyses (Pr(> |t|) = 0.035).

## Discussion

The present study compares the densities of PNNs and PV expressing neurons in the DLPFC of patients suffering from three different neuropsychiatric disorders (major depression, bipolar disorder and schizophrenia) and healthy individuals.

The distribution of PNNs and PV expressing cells in the DLPFC did not differ among the four groups of our study and it was very similar to that described before in this region (Brodmann area [BA] 9) (Beasley et al. 2002; Enwright et al. 2016; Hashimoto et al. 2003; Mauney et al. 2013; Sakai 2008). The study of Enwright et al. (2016) was focused in layer III, where, in accordance with our results, most of PV+ cells and PNNs are located, but in our present study we have also included in the analysis layers IV, V and VI. In consonance with this previous study on PV+ cells and PNNs in the DLPFC of schizophrenic patients, we have also observed that, although many PV+ cells were surrounded by PNNs, some of them (around a 25%) were devoid of these specialized extracellular matrix structures. Interestingly, no differences in this percentage were detected among the different groups analyzed. It is possible that we have failed to detect a subpopulation of PV+ cells with low levels of expression of this calcium binding protein. In fact, it has been described that, in the human DLPFC, PV+ cells surrounded by PNNs have lower immunoreactivity than those lacking these structures (Enwright et al. 2016). Some of the PNNs that were not surrounding PV+ interneurons had the typical morphology of pyramidal neurons, suggesting, as described before (Enwright et al. 2016), that a subpopulation of excitatory neurons is also enwrapped by PNNs. This is relevant for our study, since the differences in the density of PNNs that we have detected are not reflected in differences in the density of PNN specifically surrounding PV+ somata. Unfortunately, very little is known about the impact of PNN coverage on pyramidal neurons.

Regarding the density of PNNs, we have observed a trend towards a decrease in schizophrenic patients, which reaches significance if a less restrictive post hoc test is applied. This result is similar, although less dramatic, than that obtained by Mauney et al. (2013), who found a 70% reduction in this parameter. It is interesting to note that these authors improved substantially their PNN labeling when using sections from frozen samples and then postfixing them, as we have done in the present study. In a more recent study, Enwright et al. (2016) did not find changes in PNN density, but detected reduced intensities of fluorescence for PNNs markers. These results are in consonance with previous results, including our own, in mouse models of schizophrenia, which have found reductions in the number of PNNs in the medial PFC (Castillo-Gómez et al. 2017; Matuszko et al. 2017; Paylor et al. 2016).

To our knowledge only other report has studied the PNNs in the DLPFC of bipolar disorder patients and it did not find differences in their density (Mauney et al. 2013). This is apparently in contrast with our results, but this discrepancy may arise from the methodology employed to process the samples. Mauney et al. (2013) used fixed blocks of tissue to obtain their sections, while we have used sections obtained from frozen blocks and have postfixed them subsequently. This procedure improves dramatically the sensibility of the detection of PNNs, as described by the same authors (Mauney et al. 2013). Our results are in consonance with a recent study performed in the reticular thalamic nucleus, which has found decreases in the number of PNNs both in schizophrenic and bipolar patients (Steullet et al. 2018).

Our study is the first to have explored the presence of changes in the density of PNNs in the DLPFC of major depression patients, finding negative results. However, more studies need to be performed with different and larger samples of patients to discard an impact of major depression on PNNs. In fact, animals subjected to chronic stress, a model of depression, during the adolescence or during adulthood show alterations in the number of PNNs (Castillo-Gómez et al. 2017; de Araújo Costa Folha et al. 2017; Pesarico et al. 2019).

In consonance with a previous study on the BA9 of the DLPFC, which used the same collection of samples that we have analyzed in the present study (Beasley et al. 2002), we have not found differences in the density of PV immunoreactive somata in any of the patient groups. Woo et al. (1997) also failed to find differences in the density of PV+ interneurons in the BA9 of schizophrenic patients. It has to be noted, however, that other study found this density reduced in BA10 (Beasley and Reynolds 1997). Sakai (2008) also found differences in PV+ somata density in BA9 of schizophrenic patients, but only in layer IV, where their density is relatively low. A more recent study (Enwright et al. 2016) has not found significant differences in the density of PV+ cells in the layer III of BA9 of schizophrenic patients, although they discovered that the intensity of PV immunolabeling was lower in these patients than in controls. This is also in accordance with the reduced expression of PV mRNA in layers III and IV in BA9 (Hashimoto et al. 2003).

Our negative results concerning the density of PV+ cells in the DLPFC of bipolar patients are confirmatory of those obtained before in BA9 using immunohistochemistry and in situ hybridization (Beasley et al. 2002; Bitanihirwe et al. 2010; Sakai 2008). However, a study in this area using real-time quantitative PCR found a decrease in PV mRNA (Sibille et al. 2011).

We have also failed to find significant differences in the density of PV+ cells in the DLPFC of major depression patients. Similar results were found in previous reports studying the density of PV+ somata (Beasley et al. 2002; Rajkowska et al. 2007) or the expression of PV mRNA (Sibille et al. 2011). Khundakar et al. (2011) reported a decrease in the density of PV+ cells in layer III of BA9, but this study was performed in a sample of elderly major depression patients; the mean age of the group studied in the present report is younger (48 years).

To assess the influence of the demographic data on our results, we performed univariable and multivariable analyses (see “Methods” section). We report here for the first time that patients who had suffered psychotic episodes had lower PNN density. Since all of our schizophrenic patients and most of bipolar disorder patients presented psychosis during their life, we think that the lower density of PNNs may be related to the presence of psychosis per se.

Since PNNs appear to have a protective role against oxidative stress in PV expressing interneurons (Cabungcal et al. 2013), the decrease in PNN density that we have reported in psychotic individuals could be related to an increase in oxidative stress. In fact, increases in oxidative stress have been described in the brain of both SCHZ and BD patients (Andreazza et al. 2008; Yao and Keshavan 2011). Moreover, similar to what we have observed with PNNs, a previous study on the expression of reelin, an extracellular matrix protein, reported a downregulation in psychotic bipolar and schizophrenic patients when compared to control individuals and bipolar patients without psychosis (Guidotti et al. 2000). Interestingly, the presence of psychosis has been linked to abnormalities in gamma band oscillations (McNally and McCarley 2016) and on PV+ interneuron function (Sohal et al. 2009).

## Conclusion

The link between psychosis, interneurons and PNNs has to be explored in more detail, particularly using larger samples of individuals, because it may offer clues on the neurobiological bases of these symptoms in particular and on the etiopathogenesis of bipolar disorder and schizophrenia in general. In fact, several Genome Wide Association studies (GWAS) have found evidence for a common genetic basis for schizophrenia and bipolar disorder (Prata et al. 2019). Our results support this hypothesis of a common physiopathological pathway.

## Data Availability

The raw data from this study can be found online at: http://sncid.stanleyresearch.org/Default.aspx Neuropathology Consortium. Research material can be available from the authors after request.
